# Recent Research on the Role of *Lactobacilli* Probiotics in Cancer Management

**DOI:** 10.3390/nu18020297

**Published:** 2026-01-17

**Authors:** See-Hyoung Park

**Affiliations:** Department of Biological and Chemical Engineering, Hongik University, Sejong 30016, Republic of Korea; shpark74@hongik.ac.kr; Tel.: +82-44-860-2126

**Keywords:** *Lactobacilli*, probiotics, cancer, microbiome

## Abstract

*Lactobacilli* strains are one of the major groups belonging to probiotics. *Lactobacilli* strains are known to be beneficial microbes widely studied and utilized for their health benefits and applications in various fields. Recently, *Lactobacilli* strains have emerged as promising agents in cancer management due to their ability to influence various physiological processes. *Lactobacilli* strains have shown potential in producing tumor-suppressive compounds, enhancing immune responses, and reshaping gut microbiota balance for the management of various cancer types. *Lactobacilli* strains demonstrated tumor-suppressive activity through mechanisms including induction of apoptosis, inhibition of migration, and regulation of key oncogenic signaling pathways. However, the effects of *Lactobacilli* strains appear to be strain- and cancer-type-dependent, necessitating further research to identify the most effective strains for the proper cancer type with the optimal treatment regimens. In this review article, we focus on *Lactobacilli* strains studied between 2021 and 2025 that have demonstrated tumor-suppressive properties in various experimental models. In addition, this article explores the current limitations in research methodologies and proposes potential avenues for future investigations in this area of study.

## 1. Introduction

### 1.1. Probiotics

Probiotics are live microorganisms that provide health benefits when consumed in adequate amounts [1]. They are most commonly bacteria or yeast similar to those naturally found in the human gut [2]. For instance, probiotics help maintain a healthy balance of gut bacteria by suppressing harmful microbes [2]. They can support the immune system, aid digestion, and help prevent gastrointestinal disorders [3]. Probiotics are found in fermented foods such as yogurt, cheeses, and kimchi [4]. They are also available as dietary supplements in various forms such as capsules, powders, and liquids [5]. The most frequently used probiotic genera are *Lactobacillus* and *Bifidobacterium* [6]. *Lactobacillus* species produce lactic acid and help control the population of bacteria in the gut. *Bifidobacterium* species support the immune system and help break down dietary fiber. Other genera used as probiotics include *Streptococcus*, *Enterococcus*, *Saccharomyces*, and *Bacillus* [7]. Common probiotic species include *Lactobacillus acidophilus*, *Lactobacillus rhamnosus*, *Bifidobacterium longum*, and *Bifidobacterium animalis,* and each probiotic strain has unique health benefits [2]. The health benefits of probiotics depend on the specific strain and the baseline gut microbiota in each person. While probiotics are defined by the WHO as live microorganisms conferring health benefits when administered in adequate amounts, not all strains have demonstrated clinically relevant effects for specific conditions, necessitating the selection of evidence-supported candidates.

### 1.2. Health Benefits of Probiotics

A lot of health advantages have been proven for various probiotic strains. Some probiotics such as *Lactobacillus bulgaricus* and *Bifidobacterium longum* improve intestinal health by reducing symptoms of lactose intolerance by aiding lactose digestion [8]. These probiotic strains produce β-galactosidase, which digests lactose into simple sugars that can be absorbed more easily by the intestine [9]. Certain probiotics maintain cardiovascular health by regulating total and LDL cholesterol levels [10]. For example, *Lactobacillus plantarum*, *Lactobacillus rhamnosus*, and *Bifidobacterium bifidum* have been known to reduce inflammation markers such as high-sensitivity C-reactive protein and decrease total cholesterol and LDL cholesterol levels, contributing to improving vascular endothelial function [11]. Probiotics called “psychobiotics” may support mental health by influencing the gut–brain axis and producing neuroactive substances [12]. These probiotics produce several kinds of neuroactive substances such as gamma-aminobutyric acid (GABA), serotonin, catecholamines, and acetylcholine. These substances play key roles in regulating mood, stress response, and cognitive function [13]. They can influence the signaling to the brain and stimulate the enteric nervous system of the gut [13]. In addition, probiotics can reduce the incidence and severity of gastrointestinal, urinary tract, vaginal, and respiratory tract infections by modulating local microbial communities and immune responses [14]. Certain probiotics have shown promise in managing metabolic conditions such as obesity and type 2 diabetes by influencing gut microbiota and inflammatory pathways [15]. Probiotics can reduce the symptoms of allergies in susceptible individuals by activating regulatory T cells and reducing IgE-mediated allergic sensitization [16]. Probiotics contribute to the inhibition of viruses, including oncogenic viruses such as human papillomaviruses, by modulating host immune responses and interfering with viral replication [17]. Overall, while many benefits are strain-specific and require further research, probiotics are generally considered beneficial for gut, immune, and metabolic health in humans.

### 1.3. Probiotics in Cancer Management

Research on probiotics in cancer management has gained notable attention due to increasing evidence suggesting that probiotics can play a significant role in the treatment response and wellness of cancer patients [18,19]. Probiotic metabolites, particularly short-chain fatty acids (SCFAs) such as acetate, propionate, and butyrate, potentially offer protective effects against tumor development [20]. For example, butyrate improved the effectiveness of radiotherapy in organoid models derived from CRC patients. Compared to the control group, butyrate significantly promoted cell death induced by radiation by modulating the expression of the forkhead box O3A (FOXO3A) [21]. Cancer treatment often causes severe side effects and compromises the gut microbiome, leading to health problems such as infections, diarrhea, and inflammation [22]. Probiotics have shown promise in reducing the adverse effects associated with anti-tumor treatments such as chemotherapy and radiation [18]. For instance, *Lactobacillus brevis* showed a preventive effect against radiation-induced mucositis in head and neck cancer patients undergoing radiotherapy [23]. During and after treatment, probiotics have been shown to improve diverse aspects of wellness and the overall quality of life for cancer patients, including reductions in anxiety, depression, and stress as well as fewer hospitalizations due to treatment-induced complications [24]. For instance, a pilot study reported that in cancer patients with chemotherapy, *Lactobacillus rhamnosus* and *Lactobacillus helveticus* supplementation was associated with a trend toward reduced symptoms of anxiety and depression compared to the placebo group [25]. Although the potential benefits of probiotics in cancer care are promising, the research landscape is not without controversy. Some clinical trials have reported inconsistent results, raising questions about the specific strains of probiotics, dosages, and patient conditions that may influence outcomes [24,26]. As research continues to evolve, understanding the complex interactions between probiotics, gut microbiota, and cancer will be crucial for developing effective therapeutic strategies and guidelines for clinical practice.

### 1.4. Lactobacilli Probiotics

*Lactobacilli* strains are a group of Gram-positive, rod-shaped, non-spore-forming, and mostly non-motile bacteria known for producing lactic acid as their main end product from carbohydrate fermentation [27]. They are a major genus within the lactic acid bacteria family and play important roles in the natural microbiota of humans and animals, particularly in the gut, mouth, and female genital tract [28]. In addition, they have been utilized extensively in the fermentation of various foods such as yogurt, cheese, sauerkraut, and sourdough bread, which resulted in improved food preservation, flavor, texture, and inhibition of spoilage organisms [29]. Many *Lactobacillus* species, as probiotics, provide health benefits for digestion, immune modulation, infection prevention, and maintenance of a healthy microbiome balance to the human body. Some strains demonstrate health benefits like lowering cholesterol, preventing diarrhea, and potentially supporting cancer prevention or therapy [30]. Thus, *Lactobacilli* strains have been considered to exemplify a key link between traditional fermentation and modern biotechnology, valued for both food science and human health advancement. Recent taxonomic revisions have divided the *Lactobacillus* genus into multiple new genera based on genomic and ecological differences [31]. *Lactiplantibacillus*, such as *Lactiplantibacillus plantarum*, is primarily associated with diverse plant and vegetable fermentations and has high carbohydrate metabolism and various probiotic properties [32]. *Limosilactobacillus*, such as *Limosilactobacillus reuteri* and *Limosilactobacillus fermentum*, is commonly found in the intestines of humans and animals, as well as in some fermented foods, and is notable for producing antimicrobial substances [33]. In contrast, the redefined genus *Lactobacillus* is now limited to a smaller group of host-adapted species, such as *Lactobacillus delbrueckii* and *Lactobacillus acidophilus*, which are mostly associated with dairy fermentation [34]. Thus, while all three genera are lactic acid bacteria important to food and health, they differ in ecological niches, metabolic traits, and taxonomic classification due to advances in bacterial genomics.

### 1.5. Outline of Review

In this review, we comprehensively explored the emerging roles of *Lactobacilli* probiotics in cancer management, focusing on discoveries reported in the literature from 2021 to 2025. The names of the three genera (*Lactobacillus*, *Lactiplantibacillus*, and *Limosilactobacillus*) were unified and expressed as a single term (*Lactobacillus*) to avoid complicated wording and to facilitate easier understanding. We surveyed the recent literature (2015–2025) by systematically searching PubMed and Google Scholar databases using keywords for various cancer types, such as colon, cervical, gastric, breast, skin, oral, esophageal, liver, and lung, in combination with “*Lactobacillus*” or “probiotic”. This review discusses various cellular and animal models, as well as different treatment settings, by providing an extensive overview of the latest knowledge and trends on the role of probiotics in cancer management. Also, this review highlights physiological processes and therapeutic targets in diverse cancer types that may be modulated by *Lactobacilli* probiotics. Thus, this review aimed to inform future treatment strategies in cancer care and act as a valuable resource for researchers and healthcare providers.

## 2. *Lactobacilli* in Cancer

### 2.1. Lactobacilli in Colorectal Cancer

Colorectal cancer (CRC) has been the primary focus of research investigating the role of probiotics in cancer management. Studies indicate that the composition of the intestinal microbiota significantly influences CRC development [35]. An imbalance characterized by a higher prevalence of pathogenic bacteria and a lower presence of beneficial bacteria is often observed in CRC patients [36]. Probiotics have shown promise in inhibiting CRC cell growth and modulating inflammatory responses associated with tumorigenesis [37]. Research has highlighted the potential of probiotic metabolites such as SCFAs in counteracting carcinogenic processes in the colon [37]. Continued research into gut microbiome-based diagnostics and personalized therapies promises to enhance CRC management and patient survival.

Hasan reported that dried cell-free supernatant of *Lactobacillus acidophilus* isolated from homemade cow milk yogurt displayed cytotoxic effects on Caco-2 colon cancer cells [38]. The dried cell-free supernatant (0.4 mg/mL) exhibited approximately 50% inhibition of cell growth compared to the control. Apoptotic processes such as mitochondrial membrane potential and cytochrome C release were elevated, supporting the anti-tumor properties of dried cell-free supernatant in vitro.

Xu et al. reported that ^12^C^6+^ beam-induced mutant *Lactobacillus casei* suppressed tumor growth in a syngeneic (CT26) CRC mouse by reshaping gut microbiota and induction of apoptosis [39]. Mutated *Lactobacillus casei* (100:1 multiplicity of infection (MOI)) reduced the proliferation of Caco-2 cells up to about 65% compared to the wild type of *Lactobacillus casei.* Mutated *Lactobacillus casei* (1 × 10^9^ CFU (colony forming unit), 6 weeks)-fed mice showed significant changes in microbial composition and reduced tumor size up to about 83% compared to the control. This approach demonstrated the impact of engineered probiotics on tumor microenvironment regulation.

Liu et al. reported that *Lactobacillus fermentum* subspecies inhibited colon cancer by downregulating the expression of EMT (epithelial–mesenchymal transition) and Wnt/β-Catenin signaling proteins in azoxymethane/dextran sulfate sodium-induced CRC mouse [40]. *Lactobacillus fermentum* (1 × 10^11^ CFU, 13 weeks)-fed mice reduced the number and size (not quantified) of the colon tumor of the mouse compared to the non-fed control group. Western blotting and real-time polymerase chain reaction (RT-PCR) analysis indicated broad gene regulation in *Lactobacillus fermentum*-fed mice, implicating pathways critical to tumor suppression. These results support the use of targeted probiotics to modulate oncogenic pathways.

Jam et al. reported that *Lactobacillus paracasei* from the fermented dairy products in Iran demonstrated tumor-suppressive activity in dimethylhydrazine-induced CRC rats [41]. The *Lactobacillus paracasei*-fed (5 × 10^10^ CFU, 7 weeks) rat group exhibited a lower average tumor number (7.3) and average tumor size (83.5 mm^3^) than those (9.3 and 116.8 mm^3^) of the non-fed control group. The probiotic regulated cell apoptosis markers such as Bcl-2, Bax, Caspase-3, Caspase-8, and Caspase-9. This study indicates the potential of *Lactobacillus paracasei* in CRC management by proving in vivo efficacy.

An et al. reported that GABA-producing *Lactobacillus plantarum* from a biobank in Korea overcame 5-FU resistance in HT-29 CRC cells by activating GABA B receptor signaling, hindering metastatic traits, and promoting apoptosis [42]. Authors demonstrated that GABA inhibited the growth of 5FU-resistant HT-29 cells in a dose-dependent manner. Then, they showed that the dried cell-free supernatant (0.009 mg/mL) of GABA-producing *Lactobacillus plantarum* showed about 50% cytotoxicity in 5-FU resistant HT-29 compared to the control. This strategy could enhance the therapeutic efficacy of conventional chemotherapy through modulation of the novel signaling pathway in chemotherapy-resistant CRC.

Celebioglu reported that a synbiotic combination of *Lactobacillus rhamnosus* and salicylic acid decreased proliferation in HT-29 cells [43]. *Lactobacillus rhamnosus* was obtained from a laboratory in Turkey. In total, 20% of cell-free supernatants induced about 50% cytotoxicity in HT-29 cells. Synergistic action with salicylic acid resulted in elevated cytotoxicity. These results suggested promising new directions for synbiotic cancer adjuvants.

Owens et al. reported that *Lactobacillus rhamnosus* from American Type Culture Collection (ATCC) suppressed tumor development in both azoxymethane/dextran sulfate sodium-induced CRC mouse and Msh2 (MutS homolog 2) knockout CRC mouse models by modulation of CD8 T cell populations and infiltration of CD8 T cells into the tumor [44]. Supplementation of *Lactobacillus rhamnosus* (2 × 10^9^ CFU, 6 weeks) decreased the average colonic polyp number in each model from 1.5 to 0.2 and from 7 to 3. These results suggested that immunomodulatory *Lactobacillus rhamnosus* could represent a critical adjunct in CRC immunotherapy.

Yue et al. reported that *Lactobacillus acidophilus* stored in a laboratory of Northeast Agricultural University inhibited proliferation and prompted apoptosis in colon cancer cells, verifying the tumor-suppressive effects of *Lactobacillus acidophilus* in vitro [45]. Treatment of live *Lactobacillus acidophilus* (100:1 MOI) exhibited about 40% cytotoxicity in HT-29 cells, leading to downregulation of oncogenic markers and upregulation of apoptotic proteins. These findings highlighted the translational potential of *Lactobacillus acidophilus* in CRC management.

Rehman et al. reported that oral administration of *Lactobacillus acidophilus* from a microbiology bank in China altered gut microbiota and reduced tumor burden in dimethylhydrazine-induced CRC rats, indicating *Lactobacillus acidophilus*-mediated tumor-suppressive effects in vivo [46]. *Lactobacillus acidophilus* intervention (1 × 10^9^ CFU, 32 weeks) reduced tumor number in the CRC rat model up to about 35% compared to the control. This supplementation caused shifts in gut microbiota composition forward to a decrease in the harmful bacteria (*Ruminococcus obeum*, *Clostridium thermocellum*, *Bacteroides vulgates*, *Mycoplasma leachii*, and *Porphyromonas asaccharolytica*) population but increasing the beneficial bacteria (*Lactobacillus reuteri*) population. These results underline the role of gut microbial modulation in systemic CRC control.

Wang et al. reported that *Lactobacillus coryniformis* supplementation improved colitis-associated CRC outcomes by restructuring the intestinal epithelial barrier integrity and decreasing inflammation in the azoxymethane/dextran sulfate sodium-induced CRC mouse [47]. *Lactobacillus coryniformis* was isolated from a fermented *Jiangshui* vegetable in China. *Lactobacillus coryniformis* supplementation (1 × 10^9^ CFU, 14 weeks) reduced the average tumor number from 4 to 1 and restored the average colon length from 7 to 8 cm. *Lactobacillus coryniformis* fostered the restoration of microbial diversity by increasing the abundance of beneficial bacteria genera (*Lactobacillus*, *Bifidobacterium*, *Akkermansia*, and *Faecalibaculum*) but decreasing the abundance of harmful bacteria genera (*Desulfovibrio* and *Helicobacter*). These outcomes suggest therapeutic value in inflammatory CRC contexts.

Liu et al. reported that *Lactobacillus fermentum* downregulated NF-κB signaling activity, provided anti-inflammatory benefits, and reduced tumor growth in the azoxymethane/dextran sulfate sodium-induced CRC mouse [48]. *Lactobacillus fermentum* was obtained from a microbiology bank in China. Supplementation of *Lactobacillus fermentum* (1 × 10^11^ CFU, 13 weeks) reduced colon weight with lumps from 0.7 to 0.6 g. These results support the targeting of inflammation in colon cancer by *Lactobacillus fermentum* supplementation.

Sugimura et al. reported that *Lactobacillus gallinarum* from ATCC produced tumor-suppressive metabolites and favorably modulated the gut microbiome, combating colorectal tumorigenesis via metabolic and microbial mechanisms [49]. In total, 20% of cell-free supernatants induced about 100% cytotoxicity in HCT-116 cells. The author showed that *Lactobacillus gallinarum* converted L-tryptophan into indole-3-lactic acid, which showed selective cytotoxicity against HCT-116 and LoVo CRC cells while sparing normal cells. Supplementation of *Lactobacillus gallinarum* (1 × 10^8^ CFU, 12 weeks) decreased the average tumor number from 10 to 5 in azoxymethane/dextran sulfate sodium-induced CRC mice. *Lactobacillus gallinarum* increased the beneficial bacteria (*Lactobacilli* genera) population but decreased the harmful bacteria (*Alistipes*, *Allobacullum*, *Dorea*, *Odoribacter*, *Parabacteroides*, and *Ruminococcus* genera) population. This study implies that multifaceted mechanisms position *Lactobacillus gallinarum* for broader CRC management research.

Sharifi et al. reported that omega-3 combined with *Lactobacillus plantarum* synergistically reduced the proliferation of CT26 CRC cells, associated with apoptosis [50]. *Lactobacillus plantarum* was purchased from a biobank in Iran. In total, 50% of cell-free supernatants induced about 80% of cytotoxicity in CT26, which was enhanced by the cotreatment of omega-3. The mRNA levels of Bax and Caspase-3 in C26 cells were increased by cell-free supernatants, while the mRNA level of Bcl-2 was decreased at the same time. The data advocates for integrated dietary and probiotic therapies in CRC management.

Rahimpour et al. reported that *Lactobacillus rhamnosus* (R0011) boosted capecitabine efficacy against the syngeneic (CT26) CRC mouse [51]. *Lactobacillus rhamnosus* (1 × 10^8^ CFU, 6 weeks) reduced average tumor volume from 1674 to 1174 mm^3^. Enhanced protein expressions of apoptosis markers such as Bax and Caspase-3 were observed in *Lactobacillus rhamnosus*-treated tumors. These results inspire new adjunct strategies for chemotherapy for CRC.

Liotti et al. reported that *Lactobacillus rhamnosus* (LGG) from ATCC limited angiogenesis in HCT-116 cells via induction of FPR1 (formyl peptide receptor 1) activation [52]. In total, 3% of cell-free supernatants induced about a 30% wound closure effect in HCT-116 cells. VEGF-A (vascular endothelial growth factor A) expression was reduced by cell-free supernatants treatment in HCT-116, which was attenuated by shRNA against FPR1 transfection. The study demonstrates that targeting tumor vasculature with *Lactobacillus rhamnosus* could be one strategy for managing CRC.

Erfanian et al. reported that *Lactobacillus acidophilus* modulated SFRP1 (Secreted Frizzled-Related protein 1), SFRP2, and SFRP4 in HT-29 cells, leading to anti-proliferative outcomes [53]. *Lactobacillus acidophilus* was provided from a biobank in Iran. In total, 20% of cell-free supernatants showed about 80% cytotoxicity compared to the control. Wound healing assay results indicated decreased cell migration by cell-free supernatant treatment. This study indicates that *Lactobacillus acidophilus* may help treat CRC by influencing critical genes such as SFRP1, SFRP2, and SFRP4 that are involved in the Wnt signaling pathway.

Abbasi et al. reported that *Lactobacillus casei* from a biotech company in Iran showed potent cytotoxicity against HCT-116 and HT-29 cells [54]. In total, 0.1 mg/mL of dried cell-free supernatant reduced cell viability up to about 20% in both cells compared to the control. In addition, *Lactobacillus casei* supernatant induced cell cycle arrest, apoptosis, and anti-migration effects in both cells. This study showed that postbiotic metabolites from *Lactobacillus casei* demonstrated tumor-suppressive activity in colon cancer cells.

Jeong et al. reported that *Lactobacillus plantarum* from a biobank in Korea exerted tumor-suppressive effects by inhibiting autophagy and reducing the viability of Caco-2 cells [55]. In total, 10% of cell-free supernatants caused about a 50% reduction in Caco-2 cell viability compared to the control. Cell-free supernatant treatment decreased the expression of autophagy-related proteins such as ATG9A, ATG16L1, ATG5, Beclin-1, and LC3 I/II. This work supports the therapeutic targeting of CRC autophagy with *Lactobacillus plantarum*.

Vallino et al. reported that *Lactobacillus plantarum* blocked the proliferation and invasion of HT-29 and HCT-116 cells through autophagic responses [56]. *Lactobacillus plantarum* was from a biobank in Italy. In total. 1% of cell-free supernatants decreased the average cell counting number from 250,000 and 800,000 to 150,000 and 500,000 in HT-29 and HCT-116, respectively. Cell-free supernatant treatment triggered autophagy but suppressed migration and invasion in both cells. This study showed that enhanced autophagy could contribute to cancer cell clearance and underscored multifaceted tumor-suppressive mechanisms by *Lactobacillus plantarum* in CRC.

Wang et al. reported that *Lactobacillus* plantarum from Chinese pickle juice reduced colorectal tumor incidence and promoted beneficial microbiome changes in the azoxymethane/dextran sulfate sodium-induced CRC mouse [57]. Supplementation of *Lactobacillus* plantarum (1 × 10^9^ CFU, 18 weeks) decreased the average tumor number from 3.5 to 1.5 in the azoxymethane/dextran sulfate sodium-induced CRC mouse. *Lactobacillus* plantarum facilitated the repair of intestinal mucosa and decreased chronic inflammation markers. The abundance of beneficial bacteria such as *Akkermansia* was increased, but harmful bacteria such as *Parasutterella* were decreased, which was related to the recovery of SCFA production. This study supported that supplementation with *Lactobacillus* plantarum could mediate anti-CRC management by protective effects.

Salemi reported that *Lactobacillus rhamnosus* acted as a tumor-suppressive adjuvant, augmenting the chemotherapeutic effects of 5-FU and Irinotecan in Caco-2, HT-29, and HCT-116 cells [58]. *Lactobacillus rhamnosus* was provided from a biotech company in Italy. Among three cancer cells, HCT-116 was the most sensitive to cell-free supernatants. In total, 50% of cell-free supernatants caused about 50% cytotoxicity in HCT-116. This treatment led to cell cycle arrest in HT-29 and HCT-116 by increasing the G2/M population. These findings suggest the adjuvant use of *Lactobacillus rhamnosus* for optimizing current CRC therapies.

Zhou reported that *Lactobacillus fermentum* from a fermented *Jiangshui* vegetable in China alleviated colorectal tumor development in the azoxymethane/dextran sulfate sodium-induced CRC mouse [59]. In total, 10 μL of live *Lactobacillus fermentum* (OD600 = 0.6) in 90 μL of DMEM reduced cell viability up to approximately 25% in RKO and SW480 cells compared to the control. Administration of *Lactobacillus fermentum* (1 × 10^9^ CFU, 9 weeks) decreased the polyp number up to about 50% compared to the colorectal mouse model. *Lactobacillus fermentum* increased apoptosis marker levels such as p53 and Bax but decreased inflammatory marker levels such as TNF-α and IL-6. Metagenomic analyses revealed the enrichment of health-promoting genera such as *Alloprevotella* and *Lachnospireaceae* in treated animals. This outcome highlights *Lactobacillus fermentum*-based therapy prospects in CRC management by beneficially shifting gut microbiota composition and function.

Cao et al. reported that *Lactobacillus johnsonii* mediated tumor suppression in the azoxymethane/dextran sulfate sodium-induced CRC mouse under chronic stress, highlighting the interaction between probiotic effects and the host physiological state [60]. *Lactobacillus johnsonii* was provided by a biotech company in China. Administration of *Lactobacillus johnsonii* (2 × 10^8^ CFU, 10 weeks) showed under 10 mm of average tumor load in the colorectal mouse model, whereas the administration of PBS resulted in 20 mm of average tumor load. The authors found that protocatechuic acid, derived from *Lactobacillus johnsonii*, was relatively depleted in the colorectal mouse model. Subsequently, they administered protocatechuic acid to these animal models and confirmed its inhibitory effect on colon cancer. This study necessitates consideration of host factors in therapeutic interventions of *Lactobacillus johnsonii* for CRC management.

Chen et al. reported that *Lactobacillus plantarum* inhibits CRC in the azoxymethane/dextran sulfate sodium-induced CRC mouse via the modulation of gut microbial and metabolic profiles [61]. *Lactobacillus plantarum* was provided by a biotech companies in Korea. The average tumor number was observed to be 6, which was reduced to 2 by the administration of *Lactobacillus plantarum* (1 × 10^9^ CFU, 6 weeks). *Lactobacillus plantarum* reshaped the gut microbiome by increasing the abundance of beneficial genera such as *Coprococcus*, *Mucispirillum*, and *Lactobacillus* and decreasing harmful bacteria such as *Dorea*, *Shigella*, and *Alistipes*. Metabolome analysis showed that recovery was found in metabolites related to the arginine synthesis pathway by the administration of *Lactobacillus plantarum*.

Chen et al. reported that *Lactobacillus plantarum* suppressed CRC progression in the Apc (Adenomatous polyposis coli) mutant CRC mouse by promoting apoptosis through the PPARγ (peroxisome proliferator-activated receptor γ) signaling pathway [62]. *Lactobacillus plantarum* was from a biobank in China. Supplementation of *Lactobacillus plantarum* (1 × 10^9^ CFU, 10 weeks) reduced the average tumor number to about 50% compared to the control. In the *Lactobacillus plantarum*-treated group, barrier integrity and immune homeostasis improved, with concurrent downregulation of oncogenic markers. The author found that conjugated linoleic acid was the key metabolite derived from *Lactobacillus plantarum* in the suppression of CRC. According to metagenomic sequencing, *Lactobacillus plantarum* administration led to an increase in *Odoribacter splanchnicus*, potentially alleviating CRC via improvement in intestinal epithelial repair. These results show scientific significance in elucidating the tumor-suppressive role of *Lactobacillus plantarum* in the Apc mutant CRC mouse model.

Li et al. reported that *Lactobacillus reuteri* elicited tumor-suppressive responses in CT26 cells and the syngeneic (CT26) CRC mouse through apoptosis [63]. The authors did not mention the treatment concentration or CFU of *Lactobacillus reuteri*. Both *Lactobacillus reuteri* and cell-free supernatant treatment resulted in about a 50% reduction in CT26 viability. The administration of *Lactobacillus reuteri* (4 × 10^8^ CFU, 3 weeks) showed a statistically insignificant decrease in the tumor weight of the syngeneic (CT26) CRC mouse. Interestingly, the formulated microgel of *Lactobacillus reuteri* using sodium alginate and chitosan showed about a 44% reduction rate in tumor weight. Western blot analysis revealed the regulation of Bax and Bcl-2 protein expression in CRC mice. These mechanistic insights underscore the pH-resistant formulation of probiotics therapy in CRC management.

Ahrabi et al. reported that the *Lactobacillus fermentum* strain from traditional Iranian yogurt showed tumor-suppressive activity against HT-29 cells through regulation of the PTEN/PI3K/Akt pathway, which was supported by docking studies [64]. The treatment of dried cell-free supernatant (2 mg/mL) reduced cell viability up to about 50%, leading to apoptosis in HT-29 cells. In silico docking analysis confirmed the binding affinity of small molecules from dried cell-free supernatant to each potential protein target, such as PTEN, Akt, mTOR, and Caspase-3, aligning with in vitro functional results.

Recent publications collectively demonstrated the tumor-suppressive potential of various *Lactobacilli* strains against CRC (Table 1). These studies spanned in vitro cell line studies and in vivo animal model experiments. As shown in Table 1, in vitro studies adapted various human CRC cell lines, including HT-29, Caco-2, HCT-116, and LoVo, to assess the tumor-suppressive effects of different *Lactobacilli* strains. Treatment options primarily involved the application of live cells, dried bacterial cells, or cell-free supernatants with doses expressed as concentration (mg/mL), dilution ratio (%), or bacteria-to-cancer cell ratios. Treatment time ranged from 12 to 120 h. The cytotoxic assays were conducted using mostly MTT and CCK-8, providing quantitative measures of cell viability in response to *Lactobacilli* strain treatments. Several in vivo CRC animal models were employed, including the syngeneic mouse (CT26), genetically modified knockout mouse (Msh2), and chemically induced mouse models using azoxymethane/dextran sulfate sodium or dimethylhydrazine. Probiotic supplementation involved oral administration at doses ranging from 1 × 10^7^ to 1 × 10^11^ CFU per mouse, with treatment durations of 6 to 12 weeks depending on the model. The quantitative tumor-suppressive effects of *Lactobacilli* strains were mainly expressed by measuring the tumor size, tumor number, or survival rate in mouse colon cancer models. *Lactobacilli* strains exhibited direct cytotoxicity, induction of apoptosis, and inhibition of EMT on CRC cells. Some *Lactobacilli* strains, such as *acidophilus*, *casei*, *coryniformis*, *fermentum*, and *plantarum,* remodeled the gut microbiota and increased the beneficial microbial population that was associated with the inhibition of CRC progression. Specific *Lactobacilli* strains modulated the signaling pathways key to cancer progression, including Wnt/β-Catenin (relevant to EMT and cancer stemness), NF-κB, PTEN/PI3K/Akt, GABA B receptor signaling, and FPR1. Some studies assessed the anti-CRC activity of *Lactobacilli* strains in combination with salicylic acid, chemotherapeutics (5-FU), or fish oil (omega-3), showing improved efficacy. Metabolomic analyses highlighted altered SCFAs and other metabolites (indole-3-lactic acid and conjugated linoleic acid) as potential biomarkers and mediators of tumor-suppressive activity. High variability existed in tumor-suppressive efficacy depending on the strain, and *Lactobacillus plantarum* and *Lactobacillus rhamnosus* were the most extensively investigated strains. Collectively, these studies robustly demonstrate that the therapeutic implication of *Lactobacilli* strains represents a promising avenue for adjunct or preventative CRC therapy.

### 2.2. Lactobacilli in Cervical Cancer

Cervical cancer (CC) research has increasingly focused on the composition of the cervicovaginal microbiota and its role in disease development [65]. Recent studies suggest that an imbalance marked by decreased beneficial *Lactobacilli* probiotics and increased pathogenic anaerobic bacteria such as *Prevotella* and *Gardnerella* is associated with CC progression [66,67,68]. This microbial dysbiosis leads to heightened diversity and altered immune responses, including fluctuations in cytokines, increased chronic inflammation, and impaired antiviral immunity in the cervical microenvironment [69,70,71]. *Lactobacilli* probiotics show promise in restoring microbial balance, modulating local immune factors, and potentially suppressing cervical tumorigenesis [72,73]. Understanding the cervicovaginal microbiome offers new avenues for non-invasive diagnostics, prevention, and therapeutic strategies against cervical cancer.

Hu et al. reported that *Lactobacillus casei* isolated from traditional Chinese fermented foods was shown to induce apoptosis in HeLa cells by the suppression of HPV gene expression (E6/E7) [74]. The treatment of dried *Lactobacillus casei* lysates (5 mg/mL) inhibited HeLa cell proliferation up to about 50% compared to the control, leading to apoptosis in HeLa cells. Additionally, *Lactobacillus casei* lysates slowed down HeLa cell migration and altered EMT-related protein expression. These findings suggest that *Lactobacillus casei* has potential as a probiotic for cervical cancer management.

Fan et al. reported that the *Lactobacillus iners* from ATCC inhibited SiHa CC cell proliferation and migration [75]. Vaginal dysbiosis in patients showed a significant reduction in *Lactobacilli*, especially *Lactobacillus iners*. Cell-free supernatant from 10:1 MOI caused about a 70% reduction in SiHa cell viability compared to the control. KEGG analysis showed that *Lactobacillus iners* upregulated the p53 and FOXO signaling pathways but downregulated carbon metabolism in cancer.

Kirav et al. reported that *Lactobacillus plantarum* isolated from cervicovaginal flora showed antiproliferative effects on HeLa cells [76]. Dried cell-free supernatant (0.4 mg/mL) selectively targeted HeLa cells without affecting normal endothelial cells, causing up to about 90% cell death. It also modulated immune responses by decreasing pro-inflammatory TNF-α and increasing anti-inflammatory IL-10.

Pawar and Aranha reported that *Lactobacillus salivarius* from ATCC inhibited cancer cell proliferation of HeLa and SiHa cells [77]. In total, 20% of cell-free supernatant treatment exhibited about 60 and 50% cytotoxicity in HeLa and SiHa cells, respectively. This study suggested that *Lactobacillus salivarius* metabolites exerted their tumor-suppressive effect through the production of lactic acid, hydrogen peroxide, and inhibition of EMT by upregulation of E-cadherin and downregulation of MMP9.

Wan et al. reported that *Lactobacillus crispatus* from a biobank in China inhibited the proliferation and migration of Ect1/E6E7 CC cells [78]. The treatment of a 200:1 ratio (*Lactobacillus crispatus* to Ect1/E6E7 cells) reduced the proliferation rate of Ect1/E6E7 up to about 90% compared to the control, which was associated with the induction of apoptosis.

Bi et al. reported that *Lactobacillus delbrueckii* inhibited SiHa cell proliferation and promoted apoptosis by regulating the expression of apoptosis-related proteins [79]. The IC50 value for SiHa cell proliferation was observed in vitro when using dried cell lysates prepared from 4 × 10^8^ CFU *Lactobacillus delbrueckii*. In clinical trials, administering *Lactobacillus delbrueckii* vaginally during radiotherapy attenuated a reduction in *Lactobacillus* and suppressed *Streptococcus* and *Prevotella*, thereby improving the microbiota balance. The study assessed the clinical impact of *Lactobacillus delbrueckii* on vaginal microbiota dysbiosis and radiation-induced vaginal injury in gynecologic cancer patients.

Gao et al. reported that *Lactobacillus gasseri* isolated from HPV-cleared women’s cervicovaginal areas suppressed the growth of HPV-positive Ect1/E6E7 cervical cancer cells in a xenograft (Ect1/E6E7) cervical cancer zebrafish model by the regulation of innate immune responses [80]. Injecting *Lactobacillus gasseri* (1 × 10^7^ CFU) into the perivitelline space of transgenic zebrafish embryos caused tumor growth up to about 25% compared to the control. *Lactobacillus gasseri* enhanced antiviral cytokine production and might support viral clearance and CC control.

Asoudeh-Fard et al. reported that *Lactobacillus fermentum* exhibited inhibitory effects on HeLa cell proliferation and induced apoptosis via the PTEN/Akt signaling pathway [81]. The author isolated *Lactobacillus fermentum* from dairy products in Iran. Cell-free supernatant (2 ng/mL) reduced about 50% cell viability of HeLa.

He et al. reported that *Lactobacillus brevis* from the vaginas of healthy women inhibited HeLa cell proliferation [82]. An approximately 30% decrease in the proliferation of HeLa was observed in 10,000:1 MOI. Oral administration of *Lactobacillus brevis* (5 × 10^10^ CFU, 4 weeks) reduced tumor volume up to about 40% compared to the control. Western blotting analysis indicated that *Lactobacillus brevis* regulated the protein expression of the cell cycle and apoptosis in HeLa and promoted apoptosis by regulating the Akt signaling pathway.

Zhong et al. reported that *Lactobacillus crispatus* from a biobank in China suppressed SiHa cells by altering metabolic and protein expression profiles involved in tumorigenesis [83]. Co-incubation with 200:1 MOI reduced SiHa cell viability up to about 40% compared to the control. Combined analysis by the proteomic and metabolomic approaches showed the activation of ferroptosis by *Lactobacillus crispatus* in SiHa as the key mechanism.

Xu et al. reported that *Lactobacillus plantarum* from a biotech company in Korea showed tumor-suppressive effects against HPV-positive CC cells [84]. In total, 30% of cell-free supernatants inhibited the proliferation of HPV-positive HeLa and CaSki cells up to about 50% compared to the control. Meanwhile, the HPV-negative C-33A cells displayed similar growth to the control group. These results suggest that *Lactobacillus plantarum* selectively suppresses HPV-positive CC cells, underscoring a probiotic strain with targeted tumor-suppressive properties.

Recent publications demonstrated the tumor-suppressive potential of various *Lactobacilli* strains against CC (Table 2). These studies included in vitro experiments on CC cell lines such as HeLa, CaSki, SiHa, and Ect1/E6E7, assessing the effects of treatments with live bacteria or cell-free supernatants. Treatment dosages varied by bacteria-to-cancer cell ratios or dilution ratios, with exposure times ranging from 24 to 72 h. Cytotoxicity was commonly measured by assays such as MTT, XTT, and CCK-8. Two in vivo models, including the xenograft mouse (HeLa) and zebrafish (Ect1/E6E7), were used to show that *Lactobacilli* supplementation reduced tumor size. In each model, 1 × 10^7^ CFU was injected into zebrafish for 72 h and 1 × 10^11^ CFU was administered to the mouse for 4 weeks. In accordance with results observed in CRC, *Lactobacilli* strains exhibited direct cytotoxicity, induction of apoptosis, and inhibition of EMT on CC cells, as well as alteration of vaginal microbiota. Although the research results are limited, there was a report indicating that *Lactobacillus gasseri* showed tumor-suppressive effects against CC through the suppression of the NF-κB signaling pathway and the activation of the IRF3 signaling pathway. Interestingly, *Lactobacillus plantarum* reduced proliferation and induced apoptosis in HPV-positive cervical cancer cells. In contrast, non-HPV cervical cancer cells showed less responses, indicating selective tumor-suppressive effects on HPV-positive CC. Thus, recent findings support the potential of various *Lactobacilli* strains as tumor-suppressive probiotics for CC therapy.

### 2.3. Lactobacilli in Gastric Cancer

Recent research on gastric cancer (GC) has increasingly examined the role of the gastric microbiome, particularly the impact of *Lactobacilli*, on GC development [108]. Several studies observed that patients with GC often demonstrated a significant microbial imbalance compared to individuals with normal mucosa [109,110,111]. This microbial dysbiosis was characterized by both heightened microbial diversity and substantial shifts in community structure, which might contribute to immune modulation, chronic inflammation, and altered metabolic signaling within the gastric microenvironment [112,113]. *Lactobacilli* strains exhibited probiotic properties such as antagonizing pathogenic bacteria, enhancing mucosal barrier function, and exerting anti-proliferative effects against GC cells [108,109]. Thus, insights into *Lactobacilli* strains could lead to novel approaches for early diagnosis, prevention, and individualized management of GC.

Rahimia et al. reported the tumor-suppressive effect of *Lactobacillus rhamnosus* in a xenograft GC mouse model using patient-derived GC cells [85]. The authors did not describe where they obtained *Lactobacillus rhamnosus.* The tumor volume (247 mm^3^) in the untreated mice was larger than that (131 mm^3^) in *Lactobacillus rhamnosus*-supplemented (1 × 10^8^ CFU, 6 weeks) mice. The Bax protein expression was increased but Bcl2- expression was decreased by *Lactobacillus rhamnosus*. In addition, the combination of *Lactobacillus rhamnosus* and capecitabine reduced the size of the implanted GC compared to the control group, with increased apoptotic responses in the tumor tissue.

Hwang et al. reported that *Lactobacillus brevis* from kimchi in Korea induced apoptosis in AGS GC cells [86]. Heat-killed *Lactobacillus brevis* (1 × 10^9^ CFU) treatment decreased AGS cell viability up to about 41% compared to the control. However, it did not reduce the viability of human lung fibroblast MRC-5 cells. *Lactobacillus brevis* treatment led to an increased expression of pro-apoptotic markers such as Bax, Caspase-3, and Caspase-9.

Zhang et al. reported that *Lactobacillus plantarum* from a microbiology bank in China induced apoptosis in AGS cells [87]. Treatment with dried cell-free supernatant (1 mg/mL) led to a decrease in AGS cell viability up to about 70%, while GES-1 normal gastric epithelial cells were less affected. This treatment increased the expression of pro-apoptotic markers, including Bax, Bad, and Caspases-3/8/9, and decreased anti-apoptotic Bcl-2 expression.

Jauvain et al. reported that *Lactobacillus rhamnosus* mitigated gastric carcinogenesis induced by *Helicobacter pylori* infection [88]. *Lactobacillus rhamnosus* was obtained from a biotech company in France. A 10:1 MOI treatment of *Lactobacillus rhamnosus* suppressed the *Helicobacter pylori*-induced hummingbird phenotype in AGS cells up to about 40% compared to the control. Also, *Lactobacillus rhamnosus* reduced the expression of EMT markers Snail and Zeb1, prevented tight junction loss, and reduced IL-8 production in *Helicobacter pylori*-infected AGS cells.

Gupta et al. reported the role of *Lactobacillus gasseri* in disrupting the function of CagA, a key virulence factor delivered by *Helicobacter pylori* in AGS cells [89]. *Lactobacillus gasseri* was isolated from a human gastric biopsy specimen. A 100:1 MOI treatment of *Lactobacillus gasseri* decreased the hummingbird phenotype in AGS up to about 50% compared to the control. This suppression was mediated via the inhibition of CagA phosphorylation and the blocking of its interaction with SHP-2, a key oncogenic pathway in *Helicobacter pylori*-mediated GC development.

Allahverdy et al. reported that *Lactobacillus salivarius* exerted anti-proliferative and immunomodulatory effects in AGS cells [90]. By MTT assay results, an IC50 value was observed in the treatment of 10 μg/mL of cell-free supernatant in AGS cells. Cell-free supernatant treatment modified cytokine expressions, such as IL-8 and IL-10, as well as NF-κB. The overall effect was a shift toward an anti-inflammatory state in the tumor microenvironment, potentially limiting cancer progression.

Multiple *Lactobacilli* strains demonstrated tumor-suppressive activities against GC (Table 2). In most studies, the tumor-suppressive activity of *Lactobacilli* strains was evaluated using the AGS GC cell line. Standard approaches such as MTT, CCK-8, and hummingbird phenotype assay were employed as basic cytotoxicity and morphological tests. The treatment regimens varied depending on the experimental approach, including cell-free supernatant, live bacteria, and heat-killed cells with a 24 or 48 h treatment time. A study incorporating xenograft GC (patient-derived GC cells) mouse models supported the feasibility of *Lactobacillus rhamnosus* for both prevention and adjunctive treatment of GC. In that model, 1 × 10^8^ CFU was administered into a mouse for 6 weeks. *Lactobacilli* strains demonstrated direct cytotoxicity, induction of apoptosis, and inhibition of cell migration against GC cells. Interestingly, *Lactobacilli rhamnosus* and *salivarius* exerted anti-inflammatory effects, especially in the context of GC and *Helicobacter pylori* infection. Simultaneously, they modulated the tumor microenvironment and reduced pro-inflammatory responses by decreasing pathogenic cytokine production and NF-κB activity, strengthening the anti-tumor defense. Collectively, these findings underscore the growing potential for tailored *Lactobacilli* strain interventions in GC management.

### 2.4. Lactobacilli in Breast Cancer

Recent advances in breast cancer (BC) research have increasingly focused on the complex interactions between the breast and gut microbiome [114]. Several studies observed that patients with BC often showed a significant microbial imbalance compared to individuals with healthy breast tissue or benign lesions [115,116,117]. This dysbiosis can impact host immunity, drive chronic inflammation, and modulate hormone metabolism pathways, ultimately influencing the tumor microenvironment and disease outcomes [118,119]. Research also points to the presence of intratumoral microbiota, which may act as biomarkers for diagnosis and prognosis, as well as direct contributors to BC cell behavior and metastatic potential [120]. *Lactobacilli* strains are of particular interest due to their anti-BC effects [121]. For instance, regular consumption of *Lactobacillus casei* (≥4 times/week) was associated with a 35% lower BC risk in Japanese women aged from 40 to 55 [122]. Overall, a deeper understanding of the breast-associated microbiota has the potential to transform the clinical management of BC, highlighting its promise for future precision medicine applications.

Behzadi et al. reported the tumor-suppressive effects of *Lactobacillus acidophilus* from ATCC in MCF-7 BC cells and the xenograft BC (MCF-7) mouse model [91]. Dried cell-free supernatants (0.02 mg/mL) exhibited cytotoxic activity, reducing cell viability up to about 40% compared to the control, whereas they did not reduce the viability of HUVEC. The study also indicated an induction of apoptosis in MCF-7 by analyzing p53 and Bcl-2 mRNA expression. The administration of dried cell-free supernatants (2 mg/mL, 2 weeks) decreased the average tumor weight up to about 50% in the xenograft mouse model compared to the control group. The results support the potential use of *Lactobacillus acidophilus*-derived products for BC therapy.

Nasiri et al. reported *Lactobacillus brevis* isolated from regional dairy products in Iran for synergistic effects with tamoxifen on MCF-7 cells [92]. The IC50 value for MCF-7 cell viability in the MTT assay was observed at 10 mg/mL of dried cell-free supernatant. Combining dried cell-free supernatant (8 mg/mL) with tamoxifen (20 μM) enhanced cytotoxicity and induced apoptosis more effectively than either treatment alone. Tumor-suppressive effects were linked to an increased expression of Bax and downregulation of Bcl-2. The study highlighted probiotics as a strategy to augment existing chemotherapy.

Yalçınkaya et al. reported the tumor-suppressive activity of *Lactobacillus plantarum* from human feces samples in MBD-MB-231 BC cells [93]. XTT assay results showed inhibition of MDA-MB-231 growth (50%) by the treatment of dried cell lysates (5 mg/mL). Also, dried cell lysates suppressed the migration and invasion of MDA-MB-231. The authors analyzed metabolites in *Lactobacillus plantarum* by mass spectrometry and used molecular docking simulations to understand *Lactobacillus plantarum* metabolite interactions with BC targets.

Zhang et al. examined the tumor-suppressive effects of *Lactobacillus rhamnosus* from a laboratory in China [94]. *Lactobacillus rhamnosus* administration (4 × 10^9^ CFU, 3 weeks) suppressed tumor growth up to about 25% compared to the control in the xenograft BC (MBD-MB-231) mouse model. This effect was accompanied by modulation of the host gut microbiome composition. Serum metabolome changes by *Lactobacillus rhamnosus* administration indicated shifts in metabolic pathways favorable to tumor inhibition. The study suggests that *Lactobacillus rhamnosus*-targeted interventions could support BC management.

*Lactobacilli* strains exhibited tumor-suppressive activities against BC (Table 2). In vitro assessments primarily utilized MCF-7 and MDA-MB-231 BC cell lines. Common methodologies included MTT and XTT assays for cytotoxicity. Treatment modalities encompassed dried cell-free supernatants and dried cell lysates, applied at concentrations ranging from 0.02 to 10 mg/mL over 24 or 48 h. In vivo xenograft models (MDA-MB-231, 4 × 10^9^ CFU, 3 weeks) validated the translational potential of *Lactobacillus rhamnosus* with a reduction in tumor burden and alternation of intestinal microbiota. *Lactobacilli* strains showed tumor-suppressive cancer activity, such as the promotion of apoptosis and the inhibition of migration in BC cells. Collectively, these findings highlight *Lactobacilli* strains as viable adjuncts or alternatives in BC therapy.

### 2.5. Lactobacilli in Skin Cancer

Recent advances in skin cancer (SC) research have increasingly highlighted the complex interactions of the cutaneous microbiome and the host physiological system [123,124]. Several studies have shown that patients with SC exhibit marked skin microbiome dysbiosis compared with healthy individuals, characterized by shifts in key genera such as *Cutibacterium*, *Staphylococcus*, and *Corynebacterium* [125,126]. These alterations can disrupt redox balance, promote chronic inflammation, and interact with UV-induced DNA damage, ultimately shaping the local tumor microenvironment and influencing skin carcinogenesis and progression [127,128]. Probiotics, including selected *Lactobacilli* strains, are gaining attention for their potential anti-SC effects [129]. For example, lipoteichoic acid from *Lactobacillus rhamnosus* or *Lactobacillus reuteri* has been shown to delay UV-induced tumor formation, reduce melanoma incidence, and enhance anti-tumor immune responses [130,131]. Collectively, a deeper understanding of skin-associated microbiota in the context of SC may open new avenues for microbe-based adjunctive therapies in future clinical management.

Zhao et al. reported that *Lactobacillus kefiranofaciens* isolated from Tibetan kefir, a kind of fermented food, exhibited tumor-suppressive activity in the syngeneic (B16-F10) SC mouse model, which enhanced the tumor-suppressive activity of the PD-1 antibody [95]. *Lactobacillus kefiranofaciens* supplementation (1 × 10^9^ CFU) reduced average tumor volume up to about 63% compared to the control. Gut microbiota analysis revealed increased abundance of beneficial bacteria such as *Akkermansia*, *Prevotellaceae*, and *Muribaculum*. In addition, *Lactobacillus kefiranofaciens* boosted CD8+ T-cell infiltration into tumors. This study suggests *Lactobacillus kefiranofaciens* as an immunotherapy adjuvant via microbiota remodeling in controlling SC.

Bender et al. reported that *Lactobacillus reuteri* from ATCC enhanced the tumor-suppressive activity of the PD-L1 antibody in the syngeneic (B16-F10) SC mouse model [96]. Oral administration of *Lactobacillus reuteri* (1 × 10^9^ CFU, 2 weeks) reduced the average tumor volume up to about 25% compared to the control. The authors demonstrated that *Lactobacillus reuteri* metabolized tryptophan into indole-3-aldehyde, which activated the aryl hydrocarbon receptor in dendritic cells, leading to dendritic maturation and antigen presentation. This enhanced CD8+ T-cell priming and tumor-suppressive responses. This study links dietary components, microbiota, and immunotherapy response.

Li et al. reported that *Lactobacillus curvatus* from kimchi inhibited melanoma growth and migration [97]. Dried cell lysates (0.05 mg/mL) reduced B16F10 cell viability up to about 30% compared to the control. Dried cell lysates downregulated N-cadherin but upregulated E-cadherin metastatic markers, which was related to the suppression of B16F10 cell migration. In addition, tumor weight in syngeneic (B16-F10) mice was shown to be statistically reduced by intraperitoneal injection of dried cell lysates (500 mg/kg).

Budu et al. reported that *Lactobacillus plantarum* from ATCC induced apoptosis in A375 melanoma cells [98]. Live bacteria (1 × 10^10^ CFU) induced about 20% cytotoxicity in A375 cells, whereas they did not cause cytotoxicity in HaCaT, a kind of normal keratinocytes. Apoptosis in A375 was confirmed by Bax upregulation, Bcl-2 downregulation, and PARP cleavage.

*Lactobacilli*-based interventions against SC, particularly melanoma, have been explored through in vitro and in vivo approaches, revealing convergent anti-tumor mechanisms. *Lactobacilli* strain preparation for in vitro treatment included dried cell lysates and live cells. Viability in B16-F10 (mouse) or A375 (human) melanoma cells was assessed by MTT assay for 48 or 72 h. In two studies, a syngeneic (B16-F10) SC mouse model was used for evaluating the in vivo tumor-suppressive effect of *Lactobacilli* strains. *Lactobacillus kefiranofaciens* at 1 × 10^9^ CFU reduced tumor volume by approximately 63% compared to the control. *Lactobacillus reuteri* given orally at 1 × 10^9^ CFU for 2 weeks reduced tumor volume by about 25% in the presence of a PD-L1 blockade. This anti-tumor effect of *Lactobacilli* strains was associated with a reduction in viability, inhibition of migration, induction of apoptosis, and enhancement of immune checkpoint inhibitor activity. Taken together, the convergence of working mechanisms positions these strains as promising candidates for microbe-based adjunctive therapies in SC.

### 2.6. Lactobacilli in Oral Cancer

Current evidence suggests that probiotics, especially specific *Lactobacilli* strains, support oral cancer (OC) management through both direct anti-tumor effects and modulation of the oral and gut microbiome [132,133]. Experimental studies have shown that *Lactobacilli* strains could inhibit proliferation, induce apoptosis, and reduce migration and invasion in OS cell lines [134,135]. Multiple studies have reported microbiome dysbiosis in patients with OC, characterized by alterations in the genus such as *Fusobacterium*, *Porphyromonas*, and *Prevotella* when compared with healthy counterparts [136,137]. This microbial imbalance contributes to the potentiation of carcinogenic risk factors such as tobacco and alcohol, cumulatively shaping the tumor microenvironment and influencing OC progression [138]. Moreover, oral microbiome perturbations have been linked to the modulation of local immune responses, including impaired antigen presentation and altered cytokine profiles that favor tumor growth [139]. Collectively, these insights pave the way for novel microbiome-targeted interventions, highlighting probiotics as adjunctive therapies that could reshape clinical management and improve prognosis in OC patients.

Haghshenas et al. reported that the *Lactobacillus plantarum* isolated from traditional dairy products induced cytotoxicity in OSCC oral cancer cells [99]. Dried cell-free supernatant treatment (0.025 mg/mL) reduced viability up to about 70% compared to the control, with lower cytotoxicity toward normal HUVEC cells.

Fida et al. reported that *Lactobacillus plantarum* displayed a tumor-suppressive effect in Cal 27 OC cells [100]. *Lactobacillus plantarum* (100:1 MOI) treatment exerted about 50% cytotoxicity and suppression of migration in Cal 27 cells. In addition, a combination of *Lactobacillus plantarum* with 5-FU enhanced the chemotherapeutic effect compared to 5-FU alone. The treatment increased the protein expression of p53 and cleaved Caspase-3.

Nami et al. reported that *Lactobacillus plantarum* isolated from fermented milk foods in Iran showed anti-cancer properties in KB OC cells [101]. Dried cell-free supernatant (0.01 mg/mL) reduced KB cell viability up to about 30% compared to the control. However, cytotoxicity toward normal HUVEC cell lines was comparatively low for the *Lactobacillus plantarum* supernatant. Mechanistic assays indicated an induction of apoptosis with the regulation of SMAC and Survinin in KB cells treated with dried cell-free supernatant. The work highlighted traditional dairy foods as a rich source of probiotic candidates with anti-OC potential.

The same group reported a similar study [102]. Tavallaei et al. analyzed *Lactobacillus plantarum* isolated from cheese in Iran for tumor-suppressive properties in OSCC OC cells. Dried cell-free supernatant (0.025 mg/mL) showed about 50% cytotoxicity in KB cells.

Tumor-suppressive activity of *Lactobacilli* strains (in fact, *Lactobacillus plantarum*) for targeting OC has been investigated mainly via in vitro models. Establishing an in vivo oral cancer model, especially for OSCC, is difficult, as it must balance anatomical realism and technical feasibility [140]. For in vitro experiments, *Lactobacillus plantarum* was prepared as dried cell-free supernatants or viable cells. Cell viability in human KB or OSCC lines was measured using MTT assays from 6 to 72 h. The tumor-suppressive effects stemmed from decreased cell viability, suppressed migration, and promoted apoptosis. Overall, the overlapping mechanisms highlight *Lactobacillus plantarum* as a viable option for microbial adjunctive treatments in OC.

## 3. Limitations and Future Outlook

*Lactobacilli* strains demonstrated promising tumor-suppressive effects in various cancer types. Nevertheless, their application as therapeutic agents or functional food components for cancer management faces several significant limitations.

Firstly, it is necessary to consider specific formulations to deliver the *Lactobacilli* strains into the human body. For example, synbiotics (mixed encapsulation of probiotics and prebiotics) could represent an optimal formulation strategy for human delivery due to their synergistic protection and targeted release mechanisms [141,142,143]. Prebiotics such as inulin and pectin form a protective matrix around probiotics, shielding them from gastric acid, bile salts, and enzymatic degradation during gastrointestinal transit, boosting survival rates in the colon, where therapeutic action is needed [144,145]. In addition, pH-responsive coatings such as Eudragit polymers ensure site-specific release [146]. In fact, Eudragit coatings for probiotic formulations protect probiotics through gastric transit (pH 1.5–3.5) and trigger release in the colon (pH 6.5–7.5), achieving 90% bacterial viability compared to the uncoated cells [147]. Thus, the specific formulation study bridges the viability gap during gastrointestinal transit in the human body, positioning it as the standard strategy for cancer management by combining delivery efficiency with probiotic activity.

Secondly, probiotics, including *Lactobacilli* strains, could be applied to cancer management both as preventive agents and as adjuncts to therapy. With regard to prevention, probiotics aim to maintain or restore a healthy microbiota, reduce chronic inflammation, and modulate metabolism before cancer occurs [20]. In this context, probiotics are typically used as functional foods for months to years in relatively healthy or high-risk individuals. With regard to therapy, probiotics are thought to support established treatments with aims such as improving chemotherapy or immunotherapy efficacy, reducing treatment-related side-effects (mucositis, diarrhea, and infections) and modulating the tumor immune microenvironment [1]. Prevention strategies favor long-term, relatively low-risk probiotic use to shape microbiota and host physiology with a modest effect. Therapeutic use requires a more precise, personalized selection of strains, dosing, and drug combinations with potential for stronger effects but also more stringent safety requirements. Thus, preventive vs. therapeutic roles of probiotics should be clearly distinguished when designing studies or interventions, and then probiotics will be more developed as supportive and adjunctive tools.

Thirdly, research indicates that certain probiotics can inhibit tumor growth by enhancing immune function or reducing inflammation associated with cancer development [20,148,149]. Both pro-inflammatory and anti-inflammatory activities of probiotics appear to be important for managing cancer patients, but the anti-inflammatory effects seem to be emphasized more frequently as beneficial. For example, probiotics can reduce the production of pro-inflammatory mediators such as TNF-α, IL-1β, and COX-2 in cancer models, leading to the suppression of specific cancer development via the regulation of cancer-promoting chronic inflammation [150,151,152]. While less emphasized, some pro-inflammatory effects of probiotics may be beneficial by enhancing anti-tumor immune responses. Certain probiotics can stimulate the production of pro-inflammatory cytokines that activate immune cells against tumors [153,154,155]. Thus, considering strain-specific effects on the immune response, selecting specific strains or combinations may allow tailoring of the inflammatory modulation for future research. The overall immunomodulatory balance provided by probiotics seems to be key, rather than purely anti-inflammatory effects.

Fourthly, while most research has focused on gastrointestinal cancers, particularly colon cancer, there is interest in the role of probiotics in other cancer types, such as cervical, gastric, breast, skin, oral, esophageal, liver, or lung cancer [103,104,105,106,107]. However, the depth and breadth of research on the other cancer types appear to be significantly less compared to colon cancer. It appears that there is indeed a disproportionate focus on colon cancer in probiotic research related to cancer management. This is likely due to the direct interaction between probiotics and the gut microbiome, which is most relevant to cancers of the gastrointestinal tract. Lots of mechanisms by which probiotics may affect cancer (e.g., modulating the immune system, producing SCFAs, and improving gut barrier function) could potentially be relevant to other cancer types, but this has not been extensively explored in the previous publication. Given the growing understanding of the gut microbiome’s influence on overall health and systemic diseases, including cancers outside the gastrointestinal tract, there is potential for future research to explore the effects of probiotics on a wider range of cancer types.

Fifthly, the difference in approved probiotic lists across countries is an important consideration when studying the beneficial effects of probiotics as a cancer management option. Thus, there is a need for more standardized regulatory frameworks [156,157]. This cooperation can contribute to the development of international standards for probiotic approval and use. Also, moving away from the “one-size-fits-all” approach, future studies may explore personalized or precision probiotic treatments [158,159]. This involves considering the specific characteristics of the host and potentially using commensal bacteria isolated from healthy individuals’ gut microbiota. In addition, more robust clinical trials are needed to provide convincing evidence of strain-specific probiotic benefits for various health conditions. This includes addressing contradictory results in some current clinical trials, especially in cancer patients in different geographic and socioeconomic contexts. These future directions aim to advance our understanding of probiotics, improve their efficacy and safety, and ultimately translate research findings into real-world health benefits across different regulatory environments.

Sixthly, there is a difference in probiotic microbiome between humans and other experimental model animals [160]. While researchers continue to test the tumor-suppressive efficacy of probiotics through animal experiments, it is important to recognize the limitations of these studies due to the unique microbiome composition of each host. The ongoing research into probiotics’ potential tumor-suppressive effects primarily relies on animal models, which have provided valuable insights [161,162,163]. Each host, whether human or animal, possesses a distinct microbial ecosystem shaped by factors such as genetics, diet, and environment [164,165,166]. This variability poses challenges in extrapolating results from animal studies to human applications. Recent advancements in microbiome research have highlighted the complexity of host–microbe interactions and their impact on health outcomes, including cancer development and progression [167,168,169]. While animal models offer controlled experimental conditions, they may not fully capture the nuanced interplay between probiotics and the human gut ecosystem. To address these limitations, researchers are increasingly employing sophisticated techniques such as humanized mouse models and in vitro human gut simulators [170,171,172,173]. Since in vitro models inherently lack full host–microbe interactions with pH and osmolarity biases, we need to adapt more sophisticated in vitro model systems such as 3D co-culture and organoid systems in future studies. Additionally, well-designed human clinical trials are essential to validate the findings from preclinical studies on the probiotics in cancer prevention and treatment. Thus, in vitro, in vivo, and clinical experiments must be carefully designed and conducted to validate the real biological activity of the selected probiotics.

Seventhly, postbiotic and supernatant-based models offer advantages like stability and safety over live probiotics, but they have key limitations in replicating full probiotic effects. These stem from the absence of viable cells and dynamic host interactions. Live probiotics can transiently colonize the gut, adapt to host microbiomes, and produce metabolites in situ, enabling sustained modulation. Postbiotics and supernatants lack viability, so they cannot colonize or proliferate, limiting long-term effects post-administration [174]. Supernatants capture secreted metabolites but miss cell-surface components such as pili and exopolysaccharides and intracellular factors released only in vivo. Also, processing such as heat and lysis may degrade heat-labile actives, reducing potency compared to intact live cells [175]. Postbiotic composition varies by strain, growth conditions, and inactivation method, hindering reproducibility. Unlike standardized CFU for probiotics, postbiotics lack consensus dosing, complicating clinical translation [174]. Probiotics trigger multifaceted immunity via adhesion, antigen presentation, and cytokine signaling from live cells. Supernatants elicit weaker, shorter responses without ongoing stimulation, potentially insufficient for chronic conditions [175]. Therefore, the appropriate use of postbiotics and probiotics for cancer prevention and treatment requires careful consideration and continued research.

## 4. Conclusions

This review highlighted the recent challenges for managing various cancer types with *Lactobacilli* probiotics. This review examined a wide range of cellular and animal models across multiple treatment contexts, offering a comprehensive summary of current knowledge and emerging trends regarding the use of *Lactobacilli* probiotics in cancer management. This review emphasized the physiological pathways and therapeutic targets in various cancer types that can be influenced by *Lactobacilli* probiotics. Tailored *Lactobacilli* probiotic interventions can pave the way for microbiome-guided cancer prevention and therapy strategies. While recent research shows promising results for the role of *Lactobacilli* probiotics in cancer prevention and treatment, more studies are needed to fully elucidate their mechanisms of action and establish their efficacy and safety in clinical settings. Collectively, this review was intended to guide the development of future therapeutic approaches in oncology and to serve as a useful reference for both researchers and clinicians.

## Figures and Tables

**Table 1:** *Lactobacilli* probiotics in CRC management.

No.	Probiotics	Cell Model	Assay	Dose, Time ^a^	Animal Model	Dose, Time	Mechanism	Year, Ref.
**1**	*Lactobacillus acidophilus*	Caco-2	MTT	0.4 mg/mL = 0.04% (dried cell-free supernatant in RPMI), 72 h	N/A	N/A	Induction of apoptosis.	2021, [38]
**2**	*Lactobacillus casei*	Caco-2 HT-29 HCT-116	MTT	100:1 ratio (bacteria to cancer cells), 72 h	Syngeneic (CT26) CRC mouse	1 × 10^9^ CFU, 6 weeks	Induction of apoptosis. Alternation of intestinal microbiota.	2021, [39]
**3**	*Lactobacillus fermentum*	N/A	N/A	N/A	Azoxymethane/dextran sulfate sodium-induced CRC mouse	1 × 10^11^ CFU, 13 weeks	Suppression of the Wnt/β-Catenin signaling pathway.	2021, [40]
**4**	*Lactobacillus paracasei*	N/A	N/A	N/A	Dimethylhydrazine- induced CRC mouse	5 × 10^10^ CFU, 7 weeks	Induction of apoptosis.	2021, [41]
**5**	*Lactobacillus plantarum*	HT-29	WST-1	0.009 mg/mL (dried cell-free supernatant in DMEM), 48 h	N/A	N/A	Induction of apoptosis. Inhibition of migration and invasion. Suppression of the ERK/CREP pathway.	2021, [42]
**6**	*Lactobacillus rhamnosus*	HT-29	MTT	1/5 dilution = 20% = 200 μL/mL (cell-free supernatants in RPMI), 24 h	N/A	N/A	Induction of cytotoxicity.	2021, [43]
**7**	*Lactobacillus rhamnosus*	N/A	N/A	N/A	Msh2 knockout CRC mouse	2 × 10^9^ CFU, 6 weeks	Increase in CD8 T cells.	2021, [44]
**8**	*Lactobacillus acidophilus*	Caco-2 HT-29	CCK-8	100:1 ratio (bacteria to cancer cells), 48 h	N/A	N/A	Induction of apoptosis.	2022, [45]
**9**	*Lactobacillus acidophilus*	N/A	N/A	N/A	Dimethylhydrazine- induced CRC rats	1 × 10^9^ CFU, 32 weeks	Alternation of intestinal microbiota.	2022, [46]
**10**	*Lactobacillus coryniformis*	N/A	N/A	N/A	Azoxymethane/dextran sulfate sodium-induced CRC mouse	1 × 10^9^ CFU, 14 weeks	Activation of tight junction. Inhibition of inflammation. Alternation of intestinal microbiota.	2022, [47]
**11**	*Lactobacillus fermentum*	N/A	N/A	N/A	Azoxymethane/dextran sulfate sodium-induced CRC mouse	1 × 10^11^ CFU, 13 weeks	Inhibition of inflammation. Suppression of the NF-κB signaling pathway.	2022, [48]
**12**	*Lactobacillus gallinarum*	HCT-116 LoVo	MTT	1/5 dilution = 20% = 200 μL/mL (cell-free supernatants in DMEM), 120 h	Azoxymethane/dextran sulfate sodium-induced CRC mouse	1 × 10^8^ CFU, 12 weeks	Induction of apoptosis.	2022, [49]
**13**	*Lactobacillus plantarum*	CT26	MTT	1/2 dilution = 50% = 500 μL/mL (cell-free supernatants in DMEM), 24 h	N/A	N/A	Induction of apoptosis.	2022, [50]
**14**	*Lactobacillus rhamnosus*	N/A	N/A	N/A	Syngeneic (CT26) CRC mouse	1 × 10^8^ CFU, 6 weeks	Induction of apoptosis.	2022, [51]
**15**	*Lactobacillus rhamnosus*	HCT-116	Wound healing	1/30 dilution = 3.3% = 33 μL/mL (cell-free supernatants in McCoy’s 5A), 12 h	N/A	N/A	Inhibition of angiogenesis. Inhibition of migration.	2022, [52]
**16**	*Lactobacillus acidophilus*	HT-29	MTT	1/5 dilution = 20% = 200 μL/mL (cell-free supernatants in media), 72 h	N/A	N/A	Inhibition of migration. Induction of cell cycle arrest.	2023, [53]
**17**	*Lactobacillus casei*	HT-29 HCT-116	MTT	0.1 mg/mL = 0.01% (dried cell-free supernatant in RPMI), 48 h	N/A	N/A	Induction of apoptosis. Inhibition of migration.	2023, [54]
**18**	*Lactobacillus plantarum*	Caco-2	MTT	1/10 dilution = 10% = 100 μL/mL (cell-free supernatants in DMEM), 24 h	N/A	N/A	Inhibition of autophagy.	2023, [55]
**19**	*Lactiplantibacillus plantarum*	HT-29 HCT-116	Cell counting	1/100 dilution = 1% = 10 μL/mL (cell-free supernatants in DMEM), 72 h	N/A	N/A	Inhibition of migration and invasion. Induction of cell cycle arrest. Induction of autophagy.	2023, [56]
**20**	*Lactiplantibacillus plantarum*	N/A	N/A	N/A	Azoxymethane/dextran sulfate sodium-induced CRC mouse	1 × 10^9^ CFU, 18 weeks	Induction of apoptosis. Activation of tight junction. Inhibition of inflammation. Alternation of intestinal microbiota.	2023, [57]
**21**	*Lactobacillus rhamnosus*	Caco-2 HT-29 HCT-116	MTT	1/2 dilution = 50% = 500 μL/mL (cell-free supernatants in RPMI), 48 h	N/A	N/A	Induction of cell cycle arrest.	2023, [58]
**22**	*Limosilactobacillus fermentum*	RKO SW480	CCK-8	10 μL of bacteria (OD600 = 0.6) in 90 μL of DMEM, 3 h	Azoxymethane/dextran sulfate sodium-induced CRC mouse	1 × 10^9^ CFU, 9 weeks	Induction of apoptosis. Inhibition of inflammation. Alternation of intestinal microbiota.	2024, [59]
**23**	*Lactobacillus johnsonii*	N/A	N/A	N/A	Azoxymethane/dextran sulfate sodium-induced CRC mouse with chronic stress	2 × 10^8^ CFU, 10 weeks	Suppression of the cGMP/β-catenin signaling pathway.	2024, [60]
**24**	*Lactobacillus plantarum*	N/A	N/A	N/A	Azoxymethane/dextran sulfate sodium-induced CRC mouse	1 × 10^9^ CFU, 6 weeks	Alternation of intestinal bacteria.	2024, [61]
**25**	*Lactobacillus plantarum*	N/A	N/A	N/A	Apc mutant CRC mouse	1 × 10^9^ CFU, 10 weeks	Induction of apoptosis. Activation of tight junction. Inhibition of inflammation. Suppression of the NF-κB signaling pathway. Activation of PPAR-γ signaling pathway.	2024, [62]
**26**	*Lactobacillus reuteri*	CT26	CCK-8	N/A, 10 h	Syngeneic (CT26) CRC mouse	4 × 10^8^ CFU, 3 weeks	Induction of apoptosis.	2024, [63]
**27**	*Limosilactobacillus fermentum*	HT-29	MTT	2 mg/mL = 0.2% (dried cell-free supernatant in DMEM), 24 h	N/A	N/A	Induction of apoptosis.	2025, [64]

a: Most effective dose and time. CCK-8: Cell counting kit 8. MTT: 3-(4,5-dimethylthiazol-2-yl)-2,5-diphenyltetrazolium bromide. WST-1: Water-Soluble Tetrazolium 1. CFU: Colony-forming unit. N/A: Not available. In this table, the studies by each year were distinguished by shading. *Lactiplantibacillus plantarum*: Formerly *Lactobacillus plantarum*. *Limosilactobacillus fermentum*: Formerly *Lactobacillus fermentum*.

**Table 2:** *Lactobacilli* probiotics in other cancer management.

No.	Probiotics	Cell Model	Assay	Dose, Time ^a^	Animal Model	Dose, Time	Mechanism	Year, Ref.
**1**	*Lacticaseibacillus casei*	HeLa	MTT	5 mg/mL = 0.5% (dried cell lysates in DMEM), 72 h	N/A	N/A	Induction of apoptosis. Inhibition of migration.	2021, [74]
**2**	*Lactobacillus iners*	SiHa	MTT	10:1 ratio (cell-free supernatant in DMEM), 72 h	N/A	N/A	Induction of cytotoxicity. Inhibition of migration.	2021, [75]
**3**	*Lactobacillus plantarum*	HeLa	XTT	0.4 mg/mL = 0.04% (dried cell-free supernatant in RPMI), 72 h	N/A	N/A	Induction of cytotoxicity.	2022, [76]
**4**	*Lactobacillus salivarius*	HeLa SiHa	MTT	1/5 dilution = 20% = 200 μL/mL (cell-free supernatant in DMEM), 72 h	N/A	N/A	Induction of cytotoxicity.	2022, [77]
**5**	*Lactobacillus crispatu*	Ect1/E6E7	CCK-8	200:1 ratio (bacteria to cancer cells), 48 h	N/A	N/A	Induction of apoptosis. Inhibition of migration and invasion.	2023, [78]
**6**	*Lactobacillus delbrueckii*	SiHa	MTT	4 × 10^8^ CFU (dried cell lysates in DMEM), 48 h	N/A	N/A	Induction of apoptosis. Alternation of vaginal microbiota.	2023, [79]
**7**	*Lactobacillus gasseri*	N/A	N/A	N/A	Xenograft (Ect1/E6E7) cervical cancer zebrafish	1 × 10^7^ CFU, 72 h	Suppression of the NF-κB signaling pathway. Activation of the IRF3 signaling pathway.	2023, [80]
**8**	*Lactobacillus fermentum*	HeLa	MTT	2 ng/mL = 0.00002% (cell-free supernatant in DMEM), 24 h	N/A	N/A	Induction of apoptosis.	2024, [81]
**9**	*Lactobacillus brevis*	HeLa	CCK-8	10,000:1 ratio (bacteria to cancer cells), 96 h	Xenograft (Hela) cervical cancer mouse	5 × 10^10^ CFU, 4 weeks	Induction of apoptosis.	2025, [82]
**10**	*Lactobacillus crispatus*	SiHa	CCK-8	200:1 ratio (bacteria to cancer cells), 48 h	N/A	N/A	Induction of ferroptosis.	2025, [83]
**11**	*Lactiplantibacillus plantarum*	HeLa CaSki	MTT	3:10 dilution = 30% = 300 μL/mL (cell-free supernatant in DMEM), 48 h	N/A	N/A	Induction of cytotoxicity.	2025, [84]
**12**	*Lactobacillus rhamnosus*	N/A	N/A	N/A	Xenograft (patients derived gastric cancer cells) gastric cancer mouse	1 × 10^8^ CFU, 6 weeks	Induction of apoptosis. Increase in blood cell population.	2021, [85]
**13**	*Lactobacillus brevis*	AGS	MTT	1 × 10^9^ CFU (heat-killed cells in RPMI), 48 h	N/A	N/A	Induction of apoptosis.	2022, [86]
**14**	*Lactiplantibacillus plantarum*	AGS	CCK-8	0.1 mg/mL = 0.01% (dried cell-free supernatant in RPMI), 48 h	N/A	N/A	Induction of apoptosis.	2022, [87]
**15**	*Lactobacillus rhamnosus*	AGS	Cell morphology image	10:1 ratio (bacteria to cancer cells), 24 h	N/A	N/A	Suppression of hummingbird phenotype. Inhibition of migration and invasion. Activation of tight junction. Inhibition of inflammation.	2024, [88]
**16**	*Lactobacillus gasseri*	AGS	Cell morphology image	100:1 ratio (bacteria to cancer cells), 48 h	N/A	N/A	Suppression of hummingbird phenotype.	2025, [89]
**17**	*Lactobacillus salivarius*	AGS	MTT	0.01 mg/mL = 0.001% (dried cell-free supernatant in DMEM), 48 h	N/A	N/A	Induction of cytotoxicity. Inhibition of inflammation.	2025, [90]
**18**	*Lactobacillus acidophilus*	MCF-7	MTT	0.02 mg/mL = 0.002% (dried cell-free supernatant in RPMI), 48 h	Xenograft (MCF-7) breast cancer mouse	2 mg/kg (dried cell free supernatant, 2 weeks	Induction of cytotoxicity.	2021, [91]
**19**	*Lactobacillus brevis*	MCF-7	MTT	10 mg/mL = 1% (dried cell-free supernatant in RPMI), 48 h	N/A	N/A	Induction of apoptosis.	2021, [92]
**20**	*Lactobacillus plantarum*	MDA-MB-231	XTT	5 mg/mL = 0.5% (dried cell lysates in DMEM), 72 h	N/A	N/A	Induction of cytotoxicity. Inhibition of migration and invasion.	2022, [93]
**21**	*Lacticaseibacillus rhamnosus*	N/A	N/A	N/A	Xenograft (MDA-MB-231) breast cancer mouse	4 × 10^9^ CFU, 3 weeks	Alternation of intestinal microbiota.	2022, [94]
**22**	*Lactobacillus kefiranofaciens*	N/A	N/A	N/A	Syngeneic (B16-F10) skin cancer mouse	1 × 10^9^ CFU, N/A	Increase in CD8 T cells.	2022, [95]
**23**	*Lactobacillus reuteri*	N/A	N/A	N/A	Syngeneic (B16-F10) skin cancer mouse	1 × 10^9^ CFU, 2 weeks	Increase in CD4 T cells. Increase in CD8 T cells.	2023, [96]
**24**	*Latilactobacillus curvatus*	B16-F10	MTT	0.05 mg/mL = 0.005% (dried cell lysates in DMEM), 72 h	Syngeneic (B16-F10) skin cancer mouse	500 mg/kg (dried cell-free supernatant, N/A	Induction of cytotoxicity. Inhibition of migration.	2023, [97]
**25**	*Lactiplantibacillus plantarum*	A375	MTT	1 × 10^10^ CFU (live bacteria in DMEM), 48 h	N/A	N/A	Induction of apoptosis.	2024, [98]
**26**	*Lactiplantibacillus plantarum*	OSCC	MTT	0.025 mg/mL = 0.0025% (dried cell-free supernatant in EMEM), 48 h	N/A	N/A	Induction of cytotoxicity. Induction of apoptosis.	2023, [99]
**27**	*Lactiplantibacillus plantarum*	Cal27	MTT	100:1 ratio (bacteria to cancer cells), 6 h	N/A	N/A	Induction of cytotoxicity. Inhibition of migration.	2024, [100]
**28**	*Lactiplantibacillus plantarum*	KB	MTT	0.01 mg/mL = 0.001% (dried cell-free supernatant in EMEM), 48 h	N/A	N/A	Induction of cytotoxicity.	2024, [101]
**29**	*Lactiplantibacillus plantarum*	KB	MTT	0.025 mg/mL = 0.0025% (dried cell-free supernatant in EMEM), 72 h	N/A	N/A	Induction of cytotoxicity.	2025, [102]
**30**	*Lactobacillus casei*	KYSE-30	MTT	160 mg/mL = 16% (dried cell-free supernatant in RPMI), 48 h	N/A	N/A	Induction of cytotoxicity.	2022, [103]
**31**	*Lactobacillus rhamnosus*	KYSE-30	MTT	160 mg/mL = 16% (dried cell-free supernatant in RPMI), 72 h	Xenograft (KYSE-30) esophageal cancer mouse	3 mg/kg (dried cell free supernatant, 2 weeks	Induction of cytotoxicity.	2022, [104]
**32**	*Lactobacillus acidophilus*	HKCI-2 HKCI-10	MTT	1/10 dilution = 10% = 100 μL/mL (cell-free supernatant in RPMI), 48 h	Diethylnitrosamine-induced liver cancer mouse	1 × 10^9^ CFU, 28 weeks	Induction of apoptosis. Alternation of intestinal microbiota.	2024, [105]
**33**	*Lactiplantibacillus plantarum*	Murine HCC cells with *NRAS*^G12V^ oncogene	CCK-8	1 × 10^8^ CFU (live bacteria in DMEM), 48 h	N/A	N/A	Induction of senescence.	2024, [106]
**34**	*Lactobacillus rhamnosus*	N/A	N/A	N/A	Urethane-induced lung cancer mouse	1 × 10^9^ CFU, 14 weeks	Induction of mucosal humoral immunity.	2023, [107]

a: Most effective dose and time. CCK-8: Cell counting kit 8. MTT: 3-(4,5-dimethylthiazol-2-yl)-2,5-diphenyltetrazolium bromide. WST-1: Water-Soluble Tetrazolium 1. XTT: 2,3-bis-(2-methoxy-4-nitrophenyl)-5-(phenylamino) carbonyl-2H-tetrazolium hydroxide. CFU: Colony-forming unit. N/A: Not available. In the table, studies on probiotics for each type of cancer tissue were distinguished by shading. *Lactiplantibacillus plantarum:* Formerly *Lactobacillus plantarum*. *Limosilactobacillus fermentum*: Formerly *Lactobacillus fermentum. Lacticaseibacillus casei*: Formerly *Lactobacillus casei. Latilactobacillus curvatus*: Formerly *Lactobacillus curvatus*. *Lacticaseibacillus rhamnosus*: Formerly *Lactobacillus rhamnosus*.

## Data Availability

No new data were created or analyzed in this study. Data Sharing is not applicable to this article.
